# Genetic covariance between components of male reproductive success: within-pair vs. extra-pair paternity in song sparrows

**DOI:** 10.1111/jeb.12445

**Published:** 2014-09-03

**Authors:** J M Reid, P Arcese, S Losdat

**Affiliations:** * Institute of Biological and Environmental Sciences, School of Biological Sciences, University of AberdeenAberdeen, Scotland; † Department of Forest and Conservation Sciences, University of British ColumbiaVancouver, BC, Canada

**Keywords:** life-history trade-off, multiple mating, paternity success, polyandry, polygyny, quantitative genetics, reproductive strategy, sexual selection

## Abstract

The evolutionary trajectories of reproductive systems, including both male and female multiple mating and hence polygyny and polyandry, are expected to depend on the additive genetic variances and covariances in and among components of male reproductive success achieved through different reproductive tactics. However, genetic covariances among key components of male reproductive success have not been estimated in wild populations. We used comprehensive paternity data from socially monogamous but genetically polygynandrous song sparrows (*Melospiza melodia*) to estimate additive genetic variance and covariance in the total number of offspring a male sired per year outside his social pairings (i.e. his total extra-pair reproductive success achieved through multiple mating) and his liability to sire offspring produced by his socially paired female (i.e. his success in defending within-pair paternity). Both components of male fitness showed nonzero additive genetic variance, and the estimated genetic covariance was positive, implying that males with high additive genetic value for extra-pair reproduction also have high additive genetic propensity to sire their socially paired female's offspring. There was consequently no evidence of a genetic or phenotypic trade-off between male within-pair paternity success and extra-pair reproductive success. Such positive genetic covariance might be expected to facilitate ongoing evolution of polygyny and could also shape the ongoing evolution of polyandry through indirect selection.

## Introduction

In general, the evolutionary dynamics of reproductive systems and associated traits are expected to be shaped by negative and positive genetic covariances among the life-history components that define the reproductive system and hence by genetic trade-offs and synergies acting within and across the sexes (Lande, 1982; Roff, 2002). Such genetic covariances, rather than solely phenotypic covariances, therefore need to be quantified in order to understand and predict ongoing evolution (Stearns, 1989; Roff, 2002; Kruuk *et al*., 2008; Robinson & Beckerman, 2013).

One specific ambition is to understand the evolution and persistence of complex reproductive systems where males and females mate with multiple opposite-sex individuals within single reproductive episodes (termed polygyny and polyandry, respectively, Pizzari & Birkhead, 2002; Parker, 2006; Evans & Simmons, 2008; Slatyer *et al*., 2012). Polyandry has proved difficult to explain, particularly in situations where direct selection on female multiple mating appears likely to be negative (i.e. a negative female Bateman gradient beyond a single mating, Jennions & Petrie, 2000; Slatyer *et al*., 2012; Parker & Birkhead, 2013). One potential explanation is that polyandry is positively genetically correlated with components of male fitness and consequently evolves or is maintained through indirect selection (Halliday & Arnold, 1987; Keller & Reeve, 1995; Evans & Simmons, 2008; Forstmeier *et al*., 2011). In contrast, polygyny, which often occurs alongside polyandry, often seems easy to explain because multiple mating is widely expected to increase a male's total reproductive success and hence to experience positive direct selection (i.e. a positive male Bateman gradient, Halliday & Arnold, 1987; Parker, 2006; Forstmeier *et al*., 2011; Kvarnemo & Simmons, 2013). However, it is not always emphasized that these explanations for the ongoing evolution and persistence of polygyny and polyandry both depend critically on the direction and magnitude of genetic covariances among different and potentially conflicting components of male fitness (Halliday & Arnold, 1987; Evans, 2010; Kvarnemo & Simmons, 2013).

For example, one widespread but evolutionarily puzzling polygynandrous reproductive system is social monogamy with extra-pair reproduction. Here, some offspring are sired by extra-pair males rather than by a female's socially paired male, whereas the female's socially paired male commonly also sires offspring of other females with whom he is not socially paired (Jennions & Petrie, 2000; Griffith *et al*., 2002; Parker & Birkhead, 2013). More extra-pair or extra-group paternity than expected from observed parental or territorial behaviour also commonly occurs in socially polygamous populations (Jones *et al*., 2001; Kvarnemo & Simmons, 2013). Such polygynandry creates an opportunity for individual socially paired males to increase their total reproductive success through reproduction with extra-pair or extra-group females (Webster *et al*., 1995; Lebigre *et al*., 2012). However, these same males simultaneously risk losing the paternity of offspring produced by their socially paired female(s) (Westneat & Stewart, 2003; Vedder *et al*., 2011; Kvarnemo & Simmons, 2013). Substantial variation in male fitness, and consequent selection and evolution, could therefore stem from the direction and magnitude of genetic and environmental covariation in males' success in defending the paternity of their socially paired female's offspring vs. accumulating extra-pair paternity elsewhere (Webster *et al*., 1995).

Specifically, successful extra-pair sires might have relatively low within-pair paternity success due to negative genetic covariance (i.e. a genetic trade-off) between the two routes to reproductive success. Negative covariance might arise if within-pair and extra-pair reproduction imposed conflicting demands on resource allocation, for example across mate guarding vs. mate searching or across sperm allocations to within-pair vs. extra-pair matings, or if there were directly antagonistic genetic effects on such traits (Parker, 2006). Resulting trade-offs could erode male Bateman gradients and mean that the net evolutionary response to selection on male multiple mating could be small or negative, thereby negating standard evolutionary explanations for polygyny. At the same time, negative genetic covariance between male fitness components would also complicate hypotheses that explain ongoing evolution and persistence of polyandry as a function of positive genetic covariance with individual components of male fitness (e.g. Halliday & Arnold, 1987; Keller & Reeve, 1995; Evans & Simmons, 2008), because negative genetic covariance with other components of male fitness might then arise.

Conversely, successful extra-pair sires might have relatively high within-pair paternity success due to positive genetic covariance (i.e. genetic synergy) between the two routes to reproductive success. Positive covariance might arise if there were genetic variation in resource acquisition, allowing individual males to make correlated investments in within-pair and extra-pair reproduction, or if there were directly pleiotropic effects on components of both within-pair and extra-pair success such as sperm competitiveness or mating frequency. Such synergy might be expected to increase male Bateman gradients and hence facilitate evolution of both polygyny (through direct selection) and polyandry (through indirect selection, see Discussion). Quantifying the direction and magnitude of genetic covariance between male within-pair paternity and extra-pair reproduction specifically, and among other forms of ‘defensive’ vs. ‘offensive’ paternity success more generally, is therefore prerequisite to understanding the (co)evolution and persistence of multiple mating by both sexes (Evans, 2010; Fricke *et al*., 2010; Engqvist, 2011; Droge-Young *et al*., 2012; Kvarnemo & Simmons, 2013).

However, such genetic covariances have not been estimated in wild populations where males (and females) experience natural variation in reproductive success. This is due to the difficulty of accurately measuring within-pair paternity and extra-pair reproduction across sufficient males in polygynandrous populations where relatedness is sufficiently high, and measured sufficiently accurately, to support quantitative genetic analyses. It also reflects the difficulty of fitting appropriate quantitative genetic models across components of male reproductive success that have intrinsically non-Gaussian distributions, complex covariances and among-male dependencies. We used 20 years of paternity data from socially monogamous but genetically polygynandrous song sparrows (*Melospiza melodia*, Wilson) to estimate the additive genetic covariance between a male's liability to sire an offspring produced by his socially paired female (his liability for within-pair paternity success, WPPS) and his total number of extra-pair offspring sired per year (his extra-pair reproductive success, EPRS) and thereby elucidate one key genetic covariance that could shape ongoing evolution of extra-pair reproduction and underlying (co)evolution of polygyny and polyandry. We highlight methodological challenges presented by estimating this covariance and discuss the implications of estimates for understanding the (co)evolution of male and female multiple mating.

## Materials and methods

### Study system

Mandarte Island, BC, Canada (*ca*. 6 hectares), holds a resident and primarily socially monogamous song sparrow population which has been studied intensively since 1975 (Smith *et al*., 2006) and numbered *ca*. 10–50 breeding pairs during 1993–2012. Both song sparrow sexes can breed from age 1 year, with a median reproductive lifespan of 2 years (interquartile range 1–4 years, Smith *et al*., 2006; Lebigre *et al*., 2012). Pairs typically rear up to three broods of offspring during April–July each year, but females can lay up to six clutches given repeated nest failure (Smith *et al*., 2006). First and last laying dates, and hence breeding season duration, vary substantially among years (Wilson & Arcese, 2003). Females incubate clutches (typically 3–4 eggs), whereas both socially paired parents defend the breeding territory and provision hatched offspring (Smith *et al*., 2006). Both sexes can form new social pairs between years, and sometimes between breeding attempts within a single year, given mortality or divorce of their previous mate.

Each year, all nests on Mandarte were located and monitored, and all offspring surviving to *ca*. 6 days post-hatch were marked with unique combinations of metal and coloured rings to allow subsequent identification (Keller, 1998; Smith *et al*., 2006; Lebigre *et al*., 2012). All adult immigrants to Mandarte (1.1 per year on average, which is sufficient to prevent inbreeding from rapidly accumulating) were mist-netted and ringed. All adult (≥1 year old) song sparrows alive in each year were identified with annual resighting probability of *ca*. 1, meaning that all surviving individuals on Mandarte were observed in each year (Wilson *et al*., 2007). These included all socially paired adults and hence the social parents of all offspring and also included any males that remained socially unpaired due to the typically male-biased adult sex ratio (Sardell *et al*., 2010; Lebigre *et al*., 2012).

During 1993–2012, 99.6% of ringed offspring and adults were blood sampled and genotyped at 13 polymorphic microsatellite loci to allow assignment of genetic parents. Bayesian full probability models that incorporated genetic and spatial information assigned genetic sires to 99.7% of sampled offspring with ≥95% individual-level confidence (Sardell *et al*., 2010; Reid *et al*., 2014b). Moreover, paternities were subsequently verified using ≥120 polymorphic microsatellite loci and were therefore assigned with extremely high confidence. Overall, *ca*. 28% of offspring were assigned to males other than a female's observed socially paired mate and hence were classified as extra-pair offspring (Sardell *et al*., 2010; Reid *et al*., 2014b; compared with 24% in a nearby mainland song sparrow population, Hill *et al*., 2011). Genotypes of all observed mothers and offspring were congruent, confirming that mothers were correctly identified from maternal behaviour (Sardell *et al*., 2010).

For all adult males alive during 1993–2012, the genetic paternity data were used to quantify each male's observed WPPS as the number of offspring sired out of the total offspring ringed in each brood that the male reared (i.e. offspring produced by the male's socially paired female(s)). Phenotypic WPPS was therefore unobservable for socially unpaired males that did not rear any offspring and unobserved for socially paired males whose breeding attempts failed prior to offspring genotyping and paternity assignment at *ca*. 6 days post-hatch. Each male's EPRS was quantified as the total number of ringed extra-pair offspring sired per year (i.e. offspring sired in broods produced by females other than the male's socially paired mate(s)) and was observed for all adult male song sparrows alive in each year, including males that were socially unpaired. There were therefore no missing phenotypic data for EPRS or WPPS measured as numbers of ringed offspring.

### Quantitative genetic analyses

A bivariate animal model was fitted to estimate the additive genetic variances in male EPRS and liability for WPPS and the additive genetic covariance between the two. WPPS was treated as a binomial threshold trait, thereby estimating additive genetic variance in a male's underlying liability to retain rather than lose the paternity of an offspring he reared (e.g. Bennewitz *et al*., 2007; Reid *et al*., 2014a). EPRS was assumed to follow an overdispersed Poisson distribution and was not substantially zero-inflated compared with expectation given additive overdispersion. There was therefore little requirement, or power, to estimate parameters pertaining to a distinct zero-inflation process (see Reid *et al*., 2011a).

The animal model included a variance–covariance matrix of additive genetic random effects derived from pairwise kinship (*k*) coefficients calculated from pedigree data (Kruuk, 2004). As the phenotypic data spanned 1993–2012 and many males bred in multiple years (see Results), the model also included random year and individual effects on both EPRS and liability for WPPS and hence estimated year and ‘permanent individual’ (co)variances, where the latter are assumed to comprise permanent environmental and nonadditive genetic (co)variances (Kruuk, 2004). The model also included linear regressions on individual coefficient of inbreeding (*f*), thereby estimating inbreeding depression in male EPRS and liability for WPPS and ensuring that estimated additive genetic (co)variances could not be biased by unmodelled inbreeding depression (Reid & Keller, 2010). The model also included appropriate male age effects specified based on preliminary analyses, namely a linear regression of liability for WPPS on age and three-level effects on EPRS corresponding to age classes 1, 2–5 and ≥6 years.

Multivariate animal models must specify appropriate residual covariance structures to account for instantaneous random effects that influence multiple traits expressed by individuals, otherwise estimated genetic (co)variances could be biased. However, appropriate model specification can be difficult when focal traits are measured on different but overlapping and interacting timescales and sets of individuals, meaning that the form of covariance may be unknown and complex. In the current analysis, male WPPS is most usefully measured per brood rather than per year (i.e. summed across multiple broods that a male reared within a single breeding season). This is because WPPS observed per brood reflects a male's performance in defending paternity during the days when each brood was conceived and equals the observed degree of extra-pair reproduction by his socially paired female. A male's total WPPS observed per year would also incorporate among-brood variation in environmental and female effects on paternity, particularly for males that socially paired with multiple females within a single year. Variance in male liability for WPPS could then stem from among-brood rather than within-brood variation in paternity, which is less directly relevant to understanding the magnitude and mechanisms of selection on male and female multiple mating within single reproductive episodes.

In contrast, a male's total EPRS is most appropriately measured per year rather than in relation to any single breeding event, thereby quantifying a male's total annual extra-pair offspring sired and hence fitness gained through extra-pair reproduction. Breeding attempts are asynchronous across the song sparrow population within any year, because individuals vary in first clutch laying date and subsequent clutch laying dates depend on the timing and success of earlier breeding attempts (Wilson & Arcese, 2003; Smith *et al*., 2006). As is typical for populations with multibrooded life-histories, there are therefore no clearly distinct population-wide breeding events within individual years within which male EPRS could be measured.

Although only one observation of annual EPRS exists per male per year (hereafter ‘male-year’), many males reared multiple broods per year (see Results), providing multiple observations of WPPS per male-year. Residual covariance therefore cannot be simply estimated as if there were single paired observations of EPRS and WPPS within each male-year. Furthermore, for males that were socially unpaired or otherwise failed to rear any broods of offspring in a particular year, EPRS was observed but WPPS was not. Such males therefore contributed to estimates of additive genetic (co)variances in and among EPRS and WPPS with no possible residual covariance within years. We therefore fitted a model designed to robustly estimate residual covariance across all observations of WPPS and EPRS within each male-year. Specifically, we fitted random male-year effects on male liability for WPPS, thereby accounting for any correlation in WPPS observed across multiple broods reared by a male within a single year. We also fitted random male-year effects on EPRS even though, as there is exactly one observation of EPRS per male-year, male-year variance in EPRS is synonymous with residual variance. By fixing the residual variance in EPRS to a small value, we then forced all additional residual variance to be estimated as male-year variance (while allowing residual variance in male liability for WPPS to be freely estimated). Male-year covariance between EPRS and liability for WPPS was then estimable, thereby accounting for any covariance between EPRS and WPPS within male-years that was not due to additive genetic, permanent individual or year effects (Appendix S1).

Further covariances among observations of WPPS and EPRS for different males within individual years could potentially stem from numerical dependencies among these traits. Specifically, as all offspring have exactly one father, one male's EPRS will depend partly on other males' realized WPPS and vice versa, meaning that observed phenotypes are not entirely independent. However, such dependencies and any consequent biases are likely to be small in the current analysis (see Discussion and Appendix S2).

The males whose WPPS and EPRS were observed were the offspring of numerous different mothers and fathers, meaning that there was little expectation that estimated additive genetic (co)variances could be biased by common parental effects on male phenotypes. Indeed, estimated additive genetic (co)variances remained similar when random parental effects were additionally modelled (Appendix S3).

### Analysis implementation

Standard algorithms were used to compute *f*, *k* and the inverse relationship matrix from comprehensive pedigree data spanning 1975–2012 (Reid *et al*., 2011a,b, 2014b; Appendix S4). Kinship between new immigrants and existing Mandarte-hatched natives, and hence *f* of offspring of immigrant-native pairings, was defined as zero (Reid *et al*., 2006). Phenotypic data for immigrant males were excluded because *f* is undefined for immigrants (as opposed to their offspring). As additive genetic (co)variances were estimated across numerous related males whose EPRS and WPPS were observed across numerous years and females, estimates should be relatively unbiased by specific interactions between individual males and females. Interpretation may therefore be less ambiguous than in studies where phenotypes of sets of closely related males are only observed in environments posed by small numbers of females (e.g. García-González & Evans, 2010).

The animal model was fitted using Bayesian methods implemented in package MCMCglmm 2.17 in R v2.15.2 (Hadfield, 2010; R Development Core Team, 2012), using logit and log link functions for WPPS and EPRS, respectively. The model for EPRS estimated additive overdispersion as additional residual variance to that defined by the mean. Fixed effect priors were normally distributed with mean zero and large variance. The model was rerun using a variety of relatively uninformative priors on the (co)variance components and/or genetic correlation, and posterior distributions were robust to such prior variation. The pedigree was pruned to males whose EPRS and WPPS were observed and all their known ancestors. Analyses used 3005000 iterations, burn-in 5000 and thinning interval 3000, ensuring low autocorrelation among thinned samples (< 0.05). Mixing and model convergence were verified by inspecting posterior traces and by qualitative comparison and Gelman-Rubin diagnostics of posterior metrics generated from multiple independent chains.

The posterior distribution of the latent-scale heritability of male liability for WPPS was estimated as V_A_/(V_Total_ + *π*^2^/3) given logistic variance proportional to *π*^2^/3, where V_A_ is the additive genetic variance and V_Total_ is the sum of all estimated variance components (Nakagawa & Schielzeth, 2010; Reid *et al*., 2011b). The posterior distribution of the latent-scale heritability of male EPRS was estimated as V_A_/(V_Total_ + log(1/exp(x_P_) + 1)) with exp(x_P_) taken as the raw mean EPRS (Reid *et al*., 2011a). Posterior means and 95% highest posterior density credible intervals (95% CI, which are appropriate for skewed posterior distributions) for estimated effects, (co)variances, heritabilities and genetic correlations were estimated across thinned samples, as was the percentage of posterior density for the genetic covariance that exceeded zero.

Further environmental, individual or social effects that could influence WPPS or EPRS were not modelled because the current aim was to partition rather than explain phenotypic variation. Total phenotypic covariance between EPRS and liability for WPPS is not directly observable across all males, because liability for WPPS exists on an underlying scale rather than as a directly observed phenotype, and furthermore, phenotypic WPPS was not observable for all males whose EPRS was observed in each year (because some males were socially unpaired or failed to rear any offspring). However, the total covariance between EPRS and liability for WPPS can be inferred by summing all estimated covariance components. Raw means are presented ± 1SD. Data are available on Dryad.

## Results

### Distributions of EPRS and WPPS

Male EPRS, defined as the total number of ringed extra-pair offspring sired per year, was observed for 368 individual male song sparrows encompassing 892 male-years (mean 2.4 ± 1.8 years per male, range 1–10). EPRS was zero in 588 (66%) of these male-years. However, there was substantial variation, with up to 11 extra-pair offspring sired (mean 0.9, variance 2.7).

Male WPPS, defined as the number of ringed offspring that a male sired out of each brood produced by his socially paired female(s), was observed for 998 broods reared by 273 individual male song sparrows (mean 3.7 ± 3.0 broods per male, median 3, range 1–19). These 998 broods were reared across 578 male-years, comprising means of 2.1 ± 1.4 years per male (median 2, range 1–7) and 1.7 ± 0.9 (median 2, range 1–6) broods per male-year. There were therefore 578 male-years where EPRS and WPPS were both observed (comprising 256 and 322 male-years when WPPS was observed for one and multiple broods, respectively), and 314 male-years where EPRS was observed but WPPS was not (because males were socially unpaired or failed to rear any offspring). Mean brood size across all 998 observed broods was 2.8 ± 1.0 offspring (median 3, range 1–4). The mean proportion of offspring that a male sired within a brood that he reared was 0.72 ± 0.37 (range 0–1).

### Distributions of *k* and *f*

The pedigree comprising the 368 male song sparrows for whom WPPS and/or EPRS was observed and all their pedigreed ancestors totalled 671 individuals. Mean pairwise *k* was 0.058 ± 0.044 among all 671 individuals (median 0.055, range 0.000–0.471, 6% zeros) and 0.071 ± 0.037 among the 368 focal males (median 0.064, range 0.005–0.471). Mean *f* was 0.064 ± 0.053 across the 368 males (median 0.058, range 0.000–0.308).

### (Co)variances in EPRS and liability for WPPS

**Figure 1:**
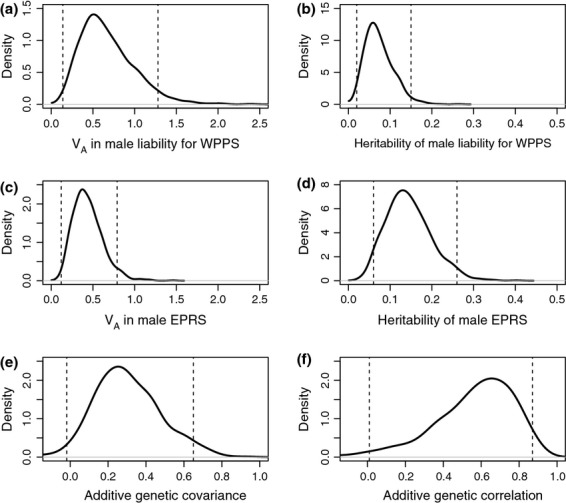
Posterior densities for (a) additive genetic variance (V_A_) and (b) heritability in male liability for within-pair paternity success (WPPS), (c) additive genetic variance and (d) heritability in male extra-pair reproductive success (EPRS) and the additive genetic (e) covariance and (f) correlation. Dashed lines demarcate 95% credible intervals.

The animal model estimated moderate additive genetic variance and heritability in both EPRS and liability for WPPS; posterior mean heritabilities were 0.14 (95% CI: 0.06–0.26) and 0.07 (95% CI: 0.02–0.15), respectively (Table1, Fig.1). The posterior mean additive genetic covariance was 0.30, equating to a posterior mean genetic correlation of 0.56 (Table1, Fig.1). Although the 95% CI for the additive genetic covariance was wide and marginally overlapped zero, 97.7% of the posterior density exceeded zero, and the 95% CI for the genetic correlation did not quite overlap zero (Table1, Fig.1). These small differences arose because the posterior distributions were slightly asymmetrical (Fig.1).

**Table 1:** Posterior mean estimates (and 95% credible intervals) for additive genetic, permanent individual, year, male-year and residual variances (V_A_, V_PI_, V_Y_, V_MY_ and V_R_, respectively), additive genetic, permanent individual, year and male-year covariances (cov_A_, cov_PI_, cov_Y_ and cov_MY_, respectively), genetic correlation (r_A_), heritability (h^2^), inbreeding depression (*β*) and age effects in male liability for within-pair paternity success (WPPS) and extra-pair reproductive success (EPRS). For EPRS, age effects are levelled at age class ≥6 years, and levels 1 and 2 show the contrasts with age classes 1 year and 2 to 5 years, respectively. For WPPS, the age effect is the regression slope. V_R_ in EPRS was fixed to 0.1 (Appendix S1)

	V_A_	cov_A_ and r_A_	V_PI_	cov_PI_	V_Y_	cov_Y_	V_MY_	cov_MY_	V_R_	Age		*β*	h^2^
WPPS	0.68 (0.14 to 1.28)	cov_A_ = 0.30 (−0.02 to 0.65)	0.45 (0.06 to 1.00)	−0.06 (−0.37 to 0.20)	0.18 (0.04 to 0.38)	−0.04 (−0.23 to 0.17)	1.01 (0.14 to 1.95)	0.43 (0.01 to 0.83)	3.57 (2.34 to 4.83)	0.14 (−0.02 to 0.27)		−1.25 (−6.76 to 4.34)	0.07 (0.02 to 0.15)
EPRS	0.44 (0.14 to 0.79)	r_A_ = 0.56 (0.01 to 0.87)	0.32 (0.08 to 0.59)		0.31 (0.07 to 0.60)		1.11 (0.75 to 1.51)		0.1 (fixed)	1: −0.92 (−1.48 to −0.30)	2: 0.68 (0.16 to 1.23)	−6.99 (−10.79 to −3.19)	0.14 (0.06 to 0.26)

The estimated permanent individual and year variances in EPRS and liability for WPPS were moderate, but the posterior mean covariances were small (Table1). The estimated male-year variances in both traits were substantial (where ‘male-year variance’ in EPRS was forced to estimate ‘residual variance’, Appendix S1), and the posterior mean male-year covariance was positive with a 95% CI that did not overlap zero (Table1). There was also substantial residual variance in liability for WPPS (Table1).

The posterior mean slope of the regression of EPRS on *f* was negative, demonstrating substantial inbreeding depression in EPRS (Table1). The regression of liability for WPPS on *f* was also negative, but the 95% CI was wide and overlapped zero (Table1). Liability for WPPS tended to increase with male age, whereas EPRS averaged higher in males aged 2–5 years and lower in 1 year old males than in males aged ≥6 years (Table1).

## Discussion

Any (co)evolution of male and female reproductive strategies is expected to depend on the direction and magnitude of genetic covariances among sex-specific life-history components, including among different components of male fitness (Jennions & Petrie, 2000; Kvarnemo & Simmons, 2013; Parker & Birkhead, 2013). Key genetic covariances include those between male reproductive success achieved through potentially conflicting reproductive tactics, where either positive or negative covariances might be predicted (Jones *et al*., 2001; House & Simmons, 2003; Parker, 2006; Evans, 2010; Fricke *et al*., 2010; Engqvist, 2011; Kvarnemo & Simmons, 2013). Empirical estimates of such covariances are therefore required, ideally across males experiencing natural variation in reproductive success.

In the context of socially monogamous but genetically polygynandrous systems, one pertinent genetic covariance is that between a male's propensity to sire an offspring produced by his socially paired female (i.e. his liability for within-pair paternity success, WPPS) and his total extra-pair reproductive success (EPRS) achieved by siring other females' offspring. The interpretation and importance of this covariance perhaps require clarification in the context of the wider literature. It does not equate to the genetic covariance between a male's liability for WPPS and his analogous liability to sire an offspring of any individual extra-pair female with whom he mates (i.e. his ‘defensive’ vs. ‘offensive’ paternity success as could be strictly defined as analogous post-copulatory traits, House & Simmons, 2003; Fricke *et al*., 2010). Nor does it equate to the genetic covariance between a male's total EPRS and his analogous total within-pair reproductive success (which depends on pairing success and female fecundity as well as WPPS, Webster *et al*., 1995). Furthermore, it does not equate to the genetic covariance between distinct precopulatory and post-copulatory episodes of sexual selection (i.e. mating success vs. subsequent fertilization success, Hosken *et al*., 2008; Droge-Young *et al*., 2012; Pischedda & Rice, 2012; Parker & Birkhead, 2013). Rather, the genetic covariance between male EPRS and liability for WPPS is of interest for three primary reasons.

First, it encapsulates the potential male trade-off between defending paternity of a socially paired female's offspring vs. achieving additional total extra-pair reproductive success elsewhere (Westneat & Stewart, 2003; Akçay *et al*., 2012; Vedder *et al*., 2011). It therefore describes the degree of evolutionary conflict or synergy across these male reproductive tactics and will influence the resulting male Bateman gradient.

Second, by affecting a male's ability to sire offspring produced by his socially paired female, a male's liability for WPPS influences his paired female's realized degree of extra-pair reproduction, whereas EPRS measures the male's reproductive success gained through extra-pair reproduction. The genetic covariance between the two components of male fitness therefore encapsulates one dimension of the potential for the observed degree of female extra-pair reproduction to evolve through genetic covariance with male extra-pair reproduction.

Third, a male's additive genetic value for EPRS can be interpreted to indicate his genetic propensity to sire any individual extra-pair offspring that is produced across a population. The genetic covariance between male EPRS and liability for WPPS will therefore shape the overall genetic covariance between female extra-pair reproduction and male liability for WPPS that could arise due to linkage disequilibria given the population-wide pattern of within-pair and extra-pair paternity. Specifically, females with high genetic value for extra-pair reproduction are by definition likely to produce extra-pair offspring with males with high genetic value for EPRS. The genetic covariance between male EPRS and liability for WPPS will therefore shape the covariance between female liability for extra-pair reproduction and male liability for WPPS that emerges across resulting offspring (Reid *et al*., 2014a). As a substantial proportion of variation in male fitness might stem from variation in WPPS (Webster *et al*., 1995; Lebigre *et al*., 2012), this covariance could facilitate ongoing evolution of female extra-pair reproduction through indirect selection.

In summary, the genetic covariance between male EPRS and liability for WPPS, as estimated here, could shape the evolutionary dynamics of both male and female extra-pair reproduction and hence of the overall socially monogamous but genetically polygynandrous reproductive system.

### Additive genetic (co)variances

Analyses of song sparrow paternity data estimated nonzero additive genetic variance in male liability for WPPS, with a latent-scale heritability of *ca*. 0.07. This implies that the paternity status of offspring within broods that a male rears, and hence the observed degree of extra-pair reproduction by the male's socially paired female, is influenced by additive genetic effects of a female's socially paired male as well as by additive genetic effects of the female herself (Reid *et al*., 2014a). The observed degree of extra-pair reproduction could therefore potentially evolve through selection on males as well as through any selection on females.

There was also nonzero additive genetic variance in male song sparrows' total annual EPRS, with an estimated heritability of *ca*. 0.14. This estimate contrasts with previous univariate analyses that did not detect such additive genetic variance in EPRS in the same song sparrow population (Reid *et al*., 2011a). This change stems from four additional years of phenotypic and pedigree data that include nonzero EPRS in related males. Evidence of nonzero heritability of male EPRS suggests one mechanism that could facilitate the evolution of female extra-pair reproduction and underlying polyandry. It implies that polyandrous females, who will by definition produce extra-pair offspring with males who are successful extra-pair sires, will on average produce extra-pair sons who are themselves relatively successful extra-pair sires, potentially creating indirect selection for polyandry and extra-pair reproduction (Wedell & Tregenza, 1999; Jennions & Petrie, 2000; Firman, 2011; Reid *et al*., 2011a; Klemme *et al*., 2014).

However, any coevolution of male and female extra-pair reproduction, and underlying polygyny and polyandry, will also depend on the additive genetic covariance between male EPRS and liability for WPPS. The estimated genetic covariance and correlation were positive in song sparrows (posterior means of 0.30 and 0.56, respectively). Although the estimated 95% CI for the genetic covariance was wide and marginally overlapped zero, more than 95% of the posterior density exceeded zero and the estimated 95% CI for the genetic correlation did not quite overlap zero. These positive estimates imply that males with high additive genetic liability to sire offspring produced by their socially paired female also had high additive genetic value for siring extra-pair offspring produced by other females. More conservatively, there was no evidence of substantial negative genetic covariance (i.e. a genetic trade-off) between the two routes to male reproductive success.

However, although the fitted animal model should adequately account for residual covariance between male EPRS and liability for WPPS within male-years (Appendix S1), some difficulties of analysis, inference and interpretation remain. Precise inference might be impeded or biased by numerical dependencies that arise because observations of EPRS and WPPS are not entirely independent across males within years (Appendix S2). Such biases might be best eliminated by fitting models that explicitly consider each individual male's liability to sire each individual offspring (rather than fitting models that consider whether or not an offspring was sired by its mother's socially paired male). However, the resulting high dimensionality is likely to render such models impractical to fit, even if restricted sets of potential sires relevant to each individual offspring were identified within the model structure. Instead, analyses of restricted data sets that minimized among-male dependencies suggested that any such biases in the current analyses are probably small (Appendix S2), supporting the conclusion that the additive genetic covariance between a male's liability to sire an offspring produced by his socially paired female and his reproductive success accrued by siring other females' offspring is most probably positive.

Positive genetic covariance between male EPRS and liability for WPPS could stem from pleiotropic genetic effects on post-copulatory and/or precopulatory processes. For example, common alleles could potentially promote success in sperm competition in the contexts of both socially paired and extra-pair females, or increase mating frequencies with both. However, in common with most field and experimental studies, EPRS and WPPS were measured across offspring that survived to paternity assignment at some point post-hatch or post-birth. Estimated genetic (co)variances might consequently reflect variation in pre-assignment offspring mortality in relation to paternity rather than variation in paternity *per se* (e.g. García-González, 2008; Droge-Young *et al*., 2012). The estimated additive genetic variance in male liability for WPPS probably does primarily reflect variation in within-pair fertilization success in song sparrows, because estimates remained quantitatively similar in univariate analyses that were restricted to breeding attempts where paternity was assigned to all conceived offspring (Reid *et al*., 2014a). In contrast, the estimated additive genetic variance in male EPRS might partly reflect genetic variation in early survival of extra-pair offspring sired by different males rather than in extra-pair mating and/or fertilization success; such effects are difficult to quantify without complete data describing paternity at conception. However, estimating the genetic covariance between male liability for WPPS and EPRS across hatched extra-pair offspring is still valuable in the context of understanding the evolutionary dynamics of extra-pair reproduction, because extra-pair offspring that die prehatch cannot contribute to female or male fitness or contribute to future correlated transmission of alleles underlying WPPS, EPRS or multiple mating.

### Environmental effects

Liability for WPPS and EPRS also showed substantial positive male-year covariance across male song sparrows (and therefore, positive total covariance calculated as the sum of all estimated covariance components, Table1). This implies that environmental effects that increased a male's liability to sire his socially paired female's offspring in a particular year also increased his success in siring extra-pair offspring in that year. Such positive covariance could stem from variation in resource acquisition and hence in ‘condition’ or attractiveness and consequent fertilization success (whether due to sperm competition or cryptic female choice) and/or mating success (Kvarnemo & Simmons, 2013). The absence of a phenotypic or genetic trade-off between male EPRS and liability for WPPS may reflect the song sparrow's multibrooded life-history and consequent local asynchrony of breeding attempts (Smith *et al*., 2006). Guarding or inseminating socially paired female(s) during their fertile period(s) might therefore not preclude males from previously or subsequently mating with fertile extra-pair females (e.g. Yezerinac & Weatherhead, 1997; Griffith *et al*., 2002; Westneat & Stewart, 2003).

### Implications and context

Positive genetic covariance between male EPRS and liability for WPPS might be predicted to facilitate evolution of male extra-pair reproduction, and underlying polygyny, because EPRS will experience both positive direct selection and positive indirect selection stemming from genetic covariance with WPPS. A positive Bateman gradient between mate and offspring numbers is likely to result (Parker & Birkhead, 2013). However, (co)evolution of absolute EPRS and WPPS must ultimately be constrained because all males within a population cannot simultaneously be both successful within-pair sires and successful extra-pair sires. Some form of soft selection and/or a genetic trade-off with some other component(s) of male or female fitness might therefore exist or arise.

Positive genetic covariance between male EPRS and liability for WPPS might also facilitate evolution of female extra-pair reproduction, and underlying polyandry, because extra-pair males with whom polyandrous females produce offspring are likely to have high additive genetic value for both EPRS (by definition) and for WPPS (due to genetic covariance). Positive genetic covariance between female propensity for polyandry and both components of male fitness could result. However, the magnitude of such cross-sex genetic covariances, and their evolutionary consequences, will also depend on the degree to which genetic covariances among female and male fitness components stem from pleiotropy vs. linkage disequilibria, and on any pattern of assortative reproduction with respect to female and male genetic values for polyandry and polygyny and associated fitness components.

Additive genetic (co)variances among male EPRS and liability for WPPS, or other broadly analogous components of male reproductive success, have not previously been explicitly estimated in wild populations. Indeed, the challenge of measuring variation in EPRS, which ideally requires paternity to be assigned to all offspring and males in a population, means that even phenotypic (co)variances between EPRS and observed WPPS have rarely been rigorously estimated (Vedder *et al*., 2011; Lebigre *et al*., 2012; see also Shuster, 2009). However, aspects of paternal behaviour and paternity varied with a chromosomal inversion (and associated colour morphs) in white-throated sparrows (*Zonotrichia albicollis*) implying that paternity success can have a genetic basis (Tuttle, 2003).

Phenotypic covariances among different components of male reproductive success, including those stemming from precopulatory vs. post-copulatory processes, have been estimated in experimental populations. Mating success and fertilization success (or associated traits) can be negatively correlated, indicating that post-copulatory sexual selection stemming from polyandry could decrease the overall opportunity for selection on male traits (Jones *et al*., 2001; Kvarnemo & Simmons, 2013). However, they can also be uncorrelated (Pischedda & Rice, 2012) or positively correlated, indicating that sequential episodes of sexual selection can be reinforcing (Droge-Young *et al*., 2012; Parker & Birkhead, 2013).

Although additive genetic variance in male paternity success can be substantial in experimental populations (Evans & Simmons, 2008; Simmons & Moore, 2009; Forstmeier *et al*., 2011), relatively few studies have explicitly estimated genetic covariances. The estimated genetic correlation between male latency to copulate and paternity success as second male was negative in *Drosophila simulans*, indicating a positive genetic correlation between male mating success and post-copulatory paternity success (Hosken *et al*., 2008). In contrast, strong negative genetic covariances among traits associated with mating success and post-copulatory fertilization success were observed in guppies (*Poecilia reticulata*), implying that the reproductive tactics of ‘courting’ and ‘sneaking’ may be genetically constrained (Evans, 2010). Negative covariance between measures of male attractiveness and nuptial provisioning or sperm viability (and hence expected fertilization success) was also observed across full-sib scorpionfly families (*Panorpa cognate*, Engqvist, 2011) and half-sib Australian cricket families (*Teleogryllus oceanicus*, Simmons *et al*., 2010). Further studies, and methodological developments, are therefore required before robust general conclusions regarding the magnitude of genetic covariances among key components of male reproductive success, the causes of such (co)variances or the consequent implications for (co)evolution of polygyny and polyandry, can be drawn.

## Supporting Information

**Appendix S1** Summary of animal model structure. **Appendix S2** Numerical dependencies among observations of EPRS and WPPS. **Appendix S3** Distributions of focal males across mothers and fathers, and maternal and paternal (co)variances. **Appendix S4** Summary of pedigree data.
