# Supplementary material for: Genetic covariance between components of male reproductive success: within-pair vs. extra-pair paternity in song sparrows
Source: J Evol Biol. 2014 Sep 3;27(10):2046–56. doi: 10.1111/jeb.12445 (PMC4283045; doi:10.1111/jeb.12445)
Supplement: Supplementary file 1 — Appendix S1 Summary of animal model structure. Appendix S2 Numerical dependencies among observations of EPRS and WPPS. Appendix S3 Distributions of focal males across mothers and fathers, and maternal and paternal (co)variances. Appendix S4 Summary of pedigree data. [file jeb0027-2046-sd1.docx]

**Supporting Information**

**Genetic covariance between components of male reproductive success: within-pair versus extra-pair paternity in song sparrows**

JANE M. REID, PETER ARCESE & SYLVAIN LOSDAT

**Appendix S1**

**Summary of animal model structure**

The two phenotypic traits of interest, a male’s extra-pair reproductive success (EPRS) and within-pair paternity success (WPPS), were measured per year and per brood respectively. Furthermore, individual males often reared multiple or zero broods within single years, providing multiple or zero observations of WPPS within male-years for which EPRS was observed. Residual covariance between EPRS and liability for WPPS therefore cannot be simply estimated across paired observations, as would be straightforward given single observations of both WPPS and EPRS per male-year.

To adequately model residual covariance, we fitted random male-year effects on both EPRS and liability for WPPS, and thereby estimated male-year variances. By fixing the residual variance in EPRS to a small value, and hence fixing the residual covariance to zero, we forced residual variance in EPRS to be estimated as male-year variance. Male-year covariance was then estimable, thereby accounting for any within-year covariance in EPRS and liability for WPPS beyond that explained by additive genetic, permanent individual and year effects. Residual variance in liability for WPPS was additionally estimated.

Example MCMCglmm code for this model is provided below.

‘number.wpo’ and ‘number.epo’ are the numbers of within-pair offspring and extra-pair offspring in observed broods respectively and ‘eprs’ is a male’s annual extra-pair reproductive success. Trait definitions and measurement are explained in the main text.

‘small’ defines the fixed value of residual variance in EPRS, which was 0.1 in the analyses presented. Posterior mean estimates of the additive genetic covariance between male EPRS and liability for WPPS were similar when the residual variance was fixed to smaller values, but model mixing was somewhat poorer.

‘fixed.effects’ denotes the set of fixed effects to be fitted (e.g. age and coefficient of inbreeding in the current analysis).

An example prior specification is provided to illustrate an appropriate structure. However, parameter values may not be appropriate for all analyses and prior sensitivity analyses should be undertaken.

*res.var <- matrix(c(1,0,0,small),2,2)*

*prior <- list(G=list(G1=list(V=diag(2),nu=1.002), G2=list(V=diag(2),nu=1.002), G3=list(V=diag(2),nu=1.002), G4=list(V=diag(2),nu=1.002)), R=list(V=res.var,nu=1.002,fix=2))*

*model.biv <- MCMCglmm(cbind(cbind(number.wpo,number.epo),eprs) ~ trait:fixed.effects,*

*random=~us(trait):animal + us(trait):individual + us(trait):year + us(trait):male.year,*

*rcov=~idh(trait):units,*

*family=c("multinomial2","poisson"), prior = prior, pedigree = pedigree, data = data …)*

**Appendix S2**

**Numerical dependencies among observations of EPRS and WPPS**

Phenotypic observations of extra-pair reproductive success (EPRS) and within-pair paternity success (WPPS) for different males within individual years are not entirely independent because some numerical dependency between WPPS and EPRS must arise across a population. Specifically, since all offspring have exactly one father, one male’s phenotypic EPRS will depend on other males’ phenotypic WPPS and vice versa. Parameter estimates, or associated confidence or credible intervals, inferred from statistical analyses that assume that all observations of WPPS and EPRS are independent could consequently be biased. However, such numerical dependencies and biases are likely to be small in the current dataset and analysis.

First, there can be no numerical dependency among observations of WPPS and EPRS within individual males within years. This is because the number of extra-pair offspring an individual male reared (i.e. offspring in broods produced by a male’s socially paired female that were sired by other males), and hence his own observed WPPS, cannot by definition contribute to the male’s own observed EPRS. Such within-male comparisons of EPRS and WPPS will contribute substantially to the estimated genetic covariance.

Second, there can be no numerical dependency among observations of WPPS and EPRS within or across males across different years because extra-pair offspring produced in any one year, and hence that contribute to estimates of WPPS in that year, can only contribute to estimates of EPRS in the same year and not in different years. Since the phenotypic observations of EPRS and WPPS used in the current analysis span 20 years, the dataset contains many more across-year comparisons of WPPS and EPRS among males than within-year comparisons, including among related males.

Third, numerical dependency was further reduced in the current analysis because phenotypic observations of WPPS and EPRS for 10 immigrant males were excluded (because the coefficient of inbreeding of these males is undefined). Some extra-pair offspring were therefore available to contribute to males’ observed EPRS that did not originate from broods that were included as observations of WPPS.

Fourth, while the expected mean male EPRS across a population (E[EPRS]) must depend on mean WPPS per brood (E[WPPS]), it also depends on the total number of broods reared across the population per year (N_broods_, which in turn depends on female population size and the number of breeding attempts per female and hence on breeding season length), on mean brood size (E[BS]) and on male population size (N_males_, including socially unpaired males that do not rear any broods), such that E[EPRS] = (1-E[WPPS]).E(BS).N_broods_/N_males_. In song sparrows, the across-year correlations between E[EPRS] and (1-E[WPPS]), E(BS), N_broods_ and N_males_ were 0.14, 0.71, 0.10 and -0.55 respectively, indicating that WPPS observed in any one individual brood may not be the primary determinant of other males’ EPRS on average. Indeed, the posterior mean year covariance between male EPRS and liability for WPPS (accounting for other random and fixed effects) was only very slightly negative with a 95%CI that substantially overlapped zero (Table 1). This shows that mean EPRS did not in fact vary markedly with mean WPPS across years.

Finally, the genetic covariance of interest involves a male’s underlying liability for WPPS rather than observed phenotypic WPPS, and any numerical dependency among observed EPRS and WPPS across males within years should not bias the posterior mean genetic covariance unless extra-pair offspring reared by one male are systematically sired by close relatives. This is not the case in Mandarte’s song sparrows (J. M. Reid unpublished data).

Although bias stemming from numerical dependency is expected to be small, further analyses were run to attempt to verify this expectation. One way to eliminate any numerical dependency between within-year observations of WPPS and EPRS, and hence eliminate any consequent bias, is to restrict the phenotypic dataset to observations of one trait within each year (i.e. EPRS or WPPS but not both). However, while this data restriction eliminates any numerical dependency, it will inevitably substantially reduce statistical power. Such data restriction therefore cannot explicitly test whether posterior means and associated 95%CIs estimated across the full dataset are biased, but can be used to examine whether overall conclusions are likely to be robust. Two further sets of analyses were therefore run.

First, an animal model with the same structure as that fitted to the full dataset (Appendix S1) was fitted to a restricted dataset that excluded phenotypic observations of EPRS from three years (1999-2001) when numerical dependencies between observations of EPRS and WPPS were likely to be greatest. Specifically, individual broods contributed <2% of all extra-pair offspring observed within a year, except that during 1999-2001 relatively small female population sizes meant that individual broods contributed *ca*.5% of all extra-pair offspring. When this relatively minor data restriction was applied, the posterior means for the additive genetic covariance and correlation between male EPRS and liability for WPPS, and the associated 95%CIs, remained quantitatively similar to those estimated from the full dataset.

Second, animal models were fitted to severely restricted datasets that excluded phenotypic observations of EPRS *or* WPPS from alternate years (dataset A, thereby completely eliminating any direct numerical dependencies), or excluded phenotypic observations of EPRS *and* WPPS from alternate years (dataset B, thereby retaining any numerical dependencies within the retained years as in the full dataset, but reducing the phenotypic dataset to a similar size as dataset A). The animal model fitted to dataset A estimated male-year variance in male liability for WPPS because multiple observations of WPPS for some male-years were still included. It also estimated independent year and residual variances for both EPRS and liability for WPPS (i.e. with zero year or residual covariance), because phenotypic observations of both traits from the same year were never included. The model fitted to dataset B had the same structure as that fitted to the full dataset (Appendix S1).

Such restrictions could be applied by selecting numerous different assortments of years. Example implementations showed that posterior means for the additive genetic covariance between male EPRS and liability for WPPS estimated from dataset A were positive, although typically slightly smaller than those estimated from the full dataset (*ca*.0.22-0.29 compared to 0.30, Table 1). As expected given the substantially reduced phenotypic dataset and hence power, the 95%CIs were wider and overlapped zero (typically *ca*.-0.2-0.75). However, models fitted to implementations of dataset B yielded similar estimates, suggesting that the wider 95%CIs primarily reflect reduced power rather than reduced bias stemming from elimination of numerical dependency.

These points and analyses suggest that the conclusion drawn from analysis of the full dataset, that the additive genetic covariance between male EPRS and liability for WPPS is most probably positive, is valid.

**Appendix S3**

**Distributions of focal males across mothers and fathers, and maternal and paternal (co)variances**

The 368 males whose EPRS was observed were the offspring of 173 different mothers and 164 different genetic fathers and were reared by 177 different social fathers. These mothers, genetic fathers and social fathers had means (±SD) of 2.1 ± 1.5 (median 2, range 1-8), 2.2 ± 1.6 (median 2, range 1-10) and 2.1 ± 1.4 (median 2, range 1-10) sons who contributed phenotypic data; 81 (47%), 71 (43%) and 87 (49%) of these parents had only one son who contributed data respectively. Furthermore the 368 males originated from 268 different mother-years, 265 genetic father-years and 271 social father-years.

The 273 males whose phenotypic WPPS was observed (i.e. that reared at least one offspring to DNA sampling and paternity assignment) were the offspring of 147 different mothers and 140 different genetic fathers and were reared by 149 different social fathers. These mothers, genetic fathers and social fathers had means of 1.9 ± 1.3 (median 1, range 1-8), 2.0 ± 1.3 (median 1.5, range 1-7) and 1.8 ± 1.2 (median 1, range 1-6) sons who contributed phenotypic data; 77 (52%), 70 (50%) and 82 (55%) of these parents had only one son who contributed data respectively. Furthermore the 273 males originated from 213 different mother-years, 209 genetic father-years and 213 social father-years.

The males for whom EPRS and WPPS were observed were therefore the offspring of numerous different mothers and genetic fathers, and were reared by numerous different social fathers, meaning that most parents had few sons who contributed data. This data structure means that there is relatively little power to distinguish parental environmental effects and (co)variances in male EPRS and liability for WPPS from permanent individual effects and (co)variances. However, it equally means that estimated additive genetic (co)variances are unlikely to be severely biased by common parental effects on the phenotypes of siblings (see Kruuk, 2004; Kruuk & Hadfield, 2007). To verify this expectation, animal models were rerun including random maternal or social paternal effects, thereby estimating (co)variances due to a focal male’s mother or social father (i.e. that reared the focal male). Posterior mean estimates for the additive genetic covariance were similar to those estimated by models that did not include these parent effects (compare Table S1 to Table 1 in the main paper). Note that small discrepancies among model estimates are expected due to Monte Carlo error. Models with both maternal and social paternal effects were not fitted because multiple mothers only produced recruited sons with one socially paired male (and vice versa).

**References**

Kruuk, L.E.B. 2004. Estimating genetic parameters in natural populations using the ‘animal model’. *Phil. Trans. R. Soc.* B **359**: 873-890.

Kruuk, L.E.B. & Hadfield, J.D. 2007. How to separate genetic and environmental causes of similarity between relatives. *J. Evol. Biol.* **20**: 1890-1903.

**Table S1.** Posterior mean estimates (and 95% highest posterior density credible intervals) for additive genetic, permanent individual, year, male-year, residual and maternal or social paternal variances (V_A_, V_PI_, V_Y_, V_MY_, V_R_ and V_Mat_ or V_Pat_ respectively), additive genetic, permanent individual, year, male-year and maternal or social paternal covariances (cov_A_, cov_PI_, cov_Y_, cov_MY_ and cov_Mat_ or cov_Pat_ respectively), genetic correlation (r_A_), heritability (h^2^), inbreeding depression (β) and age effects in male liability for within-pair paternity success (WPPS) and extra-pair reproductive success (EPRS). The percentage of the posterior density for the additive genetic covariance that exceeded zero is also shown. For EPRS, age effects are levelled at age class ≥6 years, and levels 1 and 2 show the contrasts for age classes 1 year and 2-5 years respectively. For WPPS, the age effect is the regression slope.

|  | V_A_ | cov_A_ & r_A_ | V_PI_ | cov_PI_ | V_Mat_ or V_Pat_ | cov_Mat_ or cov_Pat_ | V_Y_ | cov_Y_ | V_MY_ | cov_MY_ | V_R_ | age |  | β | h^2^ |
| --- | --- | --- | --- | --- | --- | --- | --- | --- | --- | --- | --- | --- | --- | --- | --- |
| WPPS | 0.59  (0.12-1.13) | cov_A_: 0.26  (-0.03-0.60)  96.3% | 0.38  (0.05-0.82) | -0.04  (-0.31-0.19) | V_Mat_: 0.32  (0.04-0.69) | cov_Mat_:  0.02  (-0.13-0.21) | 0.17  (0.04-0.38) | -0.03  (-0.25-0.19) | 0.97  (0.14-1.91) | 0.42  (0.07-0.86) | 3.61  (2.29-4.86) | 0.14  (-0.02-0.27) |  | -1.37  (-7.03-4.26) | 0.06  (0.02-0.13) |
| EPRS | 0.41  (0.12-0.75) | r_A_: 0.52  (-0.02-0.86) | 0.28  (0.08-0.55) |  | V_Mat_: 0.16  (0.05-0.32) |  | 0.33  (0.07-0.64) |  | 1.11  (0.76-1.50) |  | 0.1  (fixed) | 1: -0.91  (-1.47-  -0.33) | 2: 0.69  (0.16-1.18) | -7.34  (-11.16-  -3.07) | 0.13  (0.04-0.24) |
| WPPS | 0.66  (0.15-1.34) | cov_A_: 0.28  (-0.04-0.64)  97.4% | 0.40  (0.05-0.91) | -0.02  (-0.28-0.22) | V_Pat_: 0.26  (0.04-0.59) | cov_Pat_:  -0.03  (-0.21-0.15) | 0.17  (0.04-0.37) | -0.03  (-0.25-0.16) | 0.99  (0.13-1.89) | 0.45  (0.07-0.91) | 3.58  (2.43-4.85) | 0.13  (-0.03-0.27) |  | -1.20  (-6.73-3.99) | 0.07  (0.02-0.15) |
| EPRS | 0.40  (0.13-0.73) | r_A_: 0.53  (-0.02-0.86) | 0.26  (0.06-0.51) |  | V_Pat_: 0.20  (0.06-0.40) |  | 0.30  (0.07-0.61) |  | 1.10  (0.71-1.46) |  | 0.1  (fixed) | 1: -0.94  (-1.55-  -0.41) | 2: 0.66  (0.13-1.14) | -7.34  (-11.53-  -3.60) | 0.13  (0.05-0.24) |
|  |  |  |  |  |  |  |  |  |  |  |  |  |  |  |  |

**Appendix S4**

**Summary of pedigree data**

The comprehensive genetic parentage data for song sparrows hatched during 1993-2012 were used to compile a complete genetic pedigree for this period, with all individuals assigned to their most probable genetic sire (Reid et al. 2011a,b, 2014). Pedigree data derived from observed social parentage are available for 1975-1992 (Keller 1998; Reid et al. 2006, 2014). Although these data presumably contain paternity error due to unknown extra-pair paternity, all maternal links and *ca*.72% of paternal links will be correct assuming similar extra-pair reproduction rates to those observed subsequently. They therefore contain substantial information regarding relatedness among individuals that bred from 1993 onwards (and similar error to studies that use genetic pedigree data with parents assigned with 80% statistical confidence). The corrected genetic pedigree for 1993-2012 was therefore grafted onto the social pedigree for 1975-1992, thereby relaxing the alternative assumption that 1993 breeders were unrelated (Reid et al. 2011a,b). To further minimise influential pedigree error, available DNA samples were used to verify the paternity of song sparrows hatched during 1991-1992 that bred during 1993 or subsequently, including males whose EPRS and WPPS were observed (Reid et al. 2014). Since the impact of ancestral pedigree error on estimated *k* among males whose EPRS and WPPS were observed decreases rapidly with the number of intervening generations, remaining pedigree error is likely to introduce little error into animal model estimates.

**References**

Keller, L.F. 1998. Inbreeding and its fitness effects in an insular population of song sparrows (*Melospiza melodia*). *Evolution* **52**: 240-250.

Reid, J.M., Arcese, P. & Keller, L.F. 2006. Intrinsic parent-offspring correlation in inbreeding level in a song sparrow (*Melospiza melodia*) population open to immigration. *Am. Nat*. **168**: 1-13.

Reid, J.M., Arcese, P., Sardell, R.J. & Keller, L.F. 2011a. Additive genetic variance, heritability and inbreeding depression in male extra-pair reproductive success. *Am. Nat.* **177**: 177-187.

Reid, J.M., Arcese, P., Sardell, R.J. & Keller, L.F. 2011b. Heritability of female extra-pair paternity rate in song sparrows (*Melospiza melodia*). *Proc. R. Soc. B* **278**: 1114-1120.

Reid, J.M., Keller, L.F., Marr, A.B., Nietlisbach, P., Sardell, R.J. & Arcese, P. 2014. Pedigree error due to extra-pair reproduction substantially biases estimates of inbreeding depression. *Evolution* **68**: 802-815.
